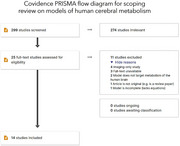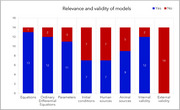# A scoping review of computational models of human cerebral metabolism

**DOI:** 10.1002/alz70855_101224

**Published:** 2025-12-23

**Authors:** Parissa Fereydouni‐Forouzandeh, Nicolas Doyon, Simon Duchesne

**Affiliations:** ^1^ Laval University, Quebec City, QC, Canada; ^2^ Quebec Heart and Lung Institute Research Centre, Quebec City, QC, Canada; ^3^ CERVO Brain Research Centre, Quebec City, QC, Canada

## Abstract

**Background:**

Metabolism is progressively downregulated below healthy performance in Alzheimer's disease, at the same time as cellular and molecular damage demand increased energy. Understanding these findings will require a multifactorial causal framework, which we propose to test via mathematical modeling (Chamberland et al., 2024). Hence, we conducted a scoping review of mathematical models of human cerebral metabolism to guide our future implementation efforts.

**Method:**

Our scoping review was conducted following the 2020 PRISMA (Preferred Reporting Items for Systematic reviews and Meta‐Analyses) guidelines. We used the following keywords while searching the PubMed database for relevant entries, from inception date to March 2024: “mathematical”, “model”, “brain”, and “glucose”. Inclusion criteria required references to be in English, original works, targeting human cerebral metabolism in humans. Abstracts and full texts were screened by two independent reviewers (PFF and SD), with data extracted by PFF. Out of 299 screened studies, 25 remained for full‐text screening, leading to 14 selected studies for data extraction and qualitative analysis (Figure 1).

**Result:**

Model replicability was overall adequate, with the inclusion of equations, parameters and initial conditions, which contributed to internal validity, while attention to the model's external validity was neglected (Figure 2). Only half of the selected studies referred to human measures in parametrization, and none performed quantitative validation with real‐life findings. In line with our desired computational approach, 12 models used ordinary differential equations. Most models focused on short timeframes, with the longer applicable window being 12 hours. The theoretical focus of the models ranged from metabolite flow between neurons and astrocytes, to depictions of glucose carriers, metabolite signaling, biophysical, and electrochemical behaviors.

**Conclusion:**

There exist computational models that appear able to capture essential components of brain metabolism on a short timeframe. Overall, these models had high internal but poor external validity. To assess the trajectory of lifetime brain metabolism, adjustments will be required.